# Branched-chain amino acid metabolism and macrophage polarization: potential regulatory nodes and therapeutic targets in diabetes-associated metabolic inflammation

**DOI:** 10.3389/fcimb.2026.1902640

**Published:** 2026-08-10

**Authors:** Ruisi Yang, Ruodi Yang, Han Zhang, Yufeng Yang, Yan Shi, Juntong Liu

**Affiliations:** 1 Department of First Clinical School, Liaoning University of Traditional Chinese Medicine, Shenyang, China; 2 Department of Academic Affairs Office, Liaoning University of Traditional Chinese Medicine, Shenyang, China; 3 School of Marxism, Liaoning University of Traditional Chinese Medicine, Shenyang, China, Shenyang, China; 4 Department of Teaching and Experiment Center, Liaoning University of Traditional Chinese Medicine, Shenyang, China

**Keywords:** branched-chain amino acid metabolism, diabetes mellitus, immunometabolic reprogramming, macrophage polarization, metabolic inflammation

## Abstract

T2DM is characterized by chronic low-grade metabolic inflammation, and imbalanced macrophage polarization is a key immunological mechanism contributing to insulin resistance (IR), pancreatic β-cell damage, and diabetes progression. Recent studies in immunometabolism suggest that BCAA metabolism regulates macrophage polarization in a context-dependent manner. Physiological BCAA catabolism supports M2-like oxidative metabolism and repair functions, whereas chronic overload of BCAA and its catabolic metabolites in diabetes may promote pro-inflammatory responses through multiple pathways. Disruptions in BCAA metabolism mediate the remodeling of macrophage immunometabolism and polarization imbalance, thereby inducing the onset and progression of metabolic inflammation in diabetes. Accordingly, this study will conduct a systematic literature search in PubMed, Web of Science, and Google Scholar to review the mechanisms by which BCAA metabolic disorders in diabetes mediate the immunometabolic reprogramming of macrophages and regulate the imbalance in M1/M2 polarization. Current evidence suggests that BCAA metabolic dysregulation can drive macrophage metabolic reprogramming through multiple pathways, leading to an imbalance in M1/M2 polarization and promoting diabetes-associated metabolic inflammation. Importantly, interventions including low-BCAA diets, caloric restriction, exercise, activators of BCAA catabolism, PPARγ agonists, berberine, ginsenoside Rb1, mulberry leaf and twig extracts, probiotics, and other gut microbiota-targeted strategies have shown potential to modulate BCAA metabolism, improve insulin sensitivity, and alleviate metabolic inflammation. Therefore, BCAA metabolism may serve as a critical link connecting metabolic dysregulation in diabetes with macrophage immunometabolic reprogramming and chronic inflammatory responses. Targeting BCAA metabolism is expected to provide new multi-target intervention strategies for metabolic inflammation in diabetes.

## 1 Introduction

In recent years, the global burden of diabetes has continued to rise. According to data from the 11th edition of the Diabetes Atlas published by the International Diabetes Federation (IDF) (Genitsaridi et al., 2026), the global prevalence of diabetes among adults aged 20–79 is projected to rise from 11.11% in 2024 to 12.96% in 2050; over the next 25 years, the global number of people with diabetes is projected to increase by approximately 44.8%. Diabetes has become a chronic metabolic disease with a continuously rising incidence worldwide, and the in-depth exploration of safe and effective intervention strategies and prevention mechanisms has become a critical issue that urgently needs to be addressed in the medical field. Although diabetes is primarily characterized by dysregulated glucose and lipid metabolism, it is also associated with chronic low-grade inflammation, immune-cell infiltration, and increased production of inflammatory mediators. Multiple lines of evidence support the classification of T2DM as a chronic metabolic disease with significant features of “metabolic inflammation” (Pradhan, 2001; Xu et al., 2003; Wang et al., 2013, 2024c). Human overfeeding studies have shown that excess nutrient intake can rapidly impair glucose utilization and insulin signaling (Boden et al., 2015). In individuals with obesity, adipose tissue expansion can lead to local hypoperfusion and hypoxia, which in turn disrupt adipokine secretion, promote macrophage recruitment and infiltration into adipose tissue, and increase the expression of inflammatory genes (Ye et al., 2007; Engin, 2017). These findings suggest that the abnormal activation of immune cells induced by metabolic stressors such as overnutrition and tissue hypoxia is a common pathological factor in the formation of the inflammatory microenvironment in diabetes. Existing clinical studies have found that while anti-inflammatory therapy alone can improve certain inflammatory markers and short-term glucose metabolism parameters, its effects are limited (Sloan-Lancaster et al., 2013; Everett et al., 2018). Although anti-inflammatory interventions can modulate certain downstream inflammatory pathways in the pathological process of diabetes, they struggle to fundamentally correct the upstream pathological network constituted by overnutrition, substrate metabolism disorders, and abnormal immune cell activation. Therefore, targeting upstream metabolic abnormalities while modulating the associated inflammatory pathways may provide a novel therapeutic strategy for the prevention and treatment of diabetes.

Macrophages are one of the most plastic cell populations in the innate immune system and are widely distributed in metabolic and immune-related tissues such as adipose tissue, the liver, blood vessel walls, the intestine, the islets of Langerhans, and joints (Gordon and Plüddemann, 2017; Locati et al., 2020). They not only participate in pathogen clearance, antigen presentation, and the amplification of inflammatory responses but also contribute to the resolution of inflammation, tissue repair, angiogenesis, and fibrosis (Sheu and Hoffmann, 2022; Siebeler et al., 2023). Depending on microenvironmental stimuli, macrophages can exhibit a continuum of functional states, with classically activated M1-like and alternatively activated M2-like states serving as widely used conceptual models (Murray et al., 2014). M1-like macrophages possess pro-inflammatory, bactericidal, and tissue damage-amplifying functions. They are typically induced by lipopolysaccharide (LPS), interferon-γ (IFN-γ), and tumor necrosis factor-α (TNF-α), and are characterized by the expression of cluster of differentiation (CD) 86, inducible nitric oxide synthase/nitric oxide synthase 2 (iNOS/NOS2), major histocompatibility complex class II molecules (MHC-II), interleukin (IL)-1β, IL-6, TNF-α, and other molecules. In contrast, M2-like macrophages are primarily induced by IL-4, IL-13, IL-10, and transforming growth factor-β (TGF-β), and are characterized by the upregulation of arginase 1 (Arg1), CD206, CD163, and IL-10. They are involved in inflammation resolution, tissue remodeling, and repair responses (Sica and Mantovani, 2012; Shapouri‐Moghaddam et al., 2018). However, macrophages in diseased tissues *in vivo* cannot be strictly classified into these two categories; rather, they form a dynamic continuum shaped by local nutrient availability, oxygen tension, lipid burden, damage-associated molecular patterns, and cytokine networks. Consequently, the M1/M2 model is understood as a simplified framework for the plasticity of macrophage function (Murray et al., 2014; Viola et al., 2019). Multiple studies have found (Eguchi and Nagai, 2017; Zhang et al., 2025a) that the diabetic microenvironment can induce a shift of macrophages toward the M1 phenotype, exacerbating inflammatory responses and driving IR and damage to pancreatic β-cells.

Immunometabolic reprogramming has emerged as a central frontier in the interdisciplinary field of immunology, metabolic medicine, and inflammatory biology in recent years (Mathis and Shoelson, 2011). While cellular metabolism primarily supplies immune cells with ATP and biosynthetic substrates, immunometabolism has further revealed that metabolic pathways, enzymes, and intermediates themselves can directly regulate immune cell differentiation, polarization, cytokine expression, and epigenetic states (Jha et al., 2015; Lee, 2026). Macrophage polarization is closely coupled with glycolysis, tricarboxylic acid (TCA) cycle, oxidative phosphorylation (OXPHOS), fatty acid metabolism, amino acid metabolism, and mitochondrial function (O’Neill et al., 2016). BCAAs, including leucine, isoleucine, and valine, are important metabolic substrates whose catabolic pathways, associated enzymes, and metabolic intermediates can influence immunometabolic reprogramming. Dysregulation of BCAA metabolism may therefore contribute to the regulation of macrophage activation and phenotypic and functional transitions (Mehla and Singh, 2019; Lu et al., 2024). Studies have shown that leucine catabolism yields end products such as acetyl-CoA and acetoacetate. By replenishing the TCA cycle and regulating the levels of metabolic intermediates such as α-ketoglutarate (α-KG), these processes reshape macrophage metabolic flux, inflammatory signaling, and epigenetic status, thereby reprogramming their activation process (Hutson et al., 2001). Elevated circulating BCAA levels are considered an early and potentially predictive metabolic hallmark of diabetes (Vanweert et al., 2022). High levels of BCAAs promote M1 polarization of macrophages and increase pro-inflammatory cytokines in adipose tissue, thereby leading to IR (Huang et al., 2024). Thus, BCAA dysregulation often precedes IR and serves as a key upstream initiator of immune-metabolic imbalance in the body. In the pathological microenvironment of diabetes, factors such as nutritional excess and tissue hypoxia can further exacerbate BCAA catabolic dysfunction, leading to the persistent accumulation of BCAA and their derivatives. The resulting metabolic disturbance may induce macrophage immunometabolic reprogramming, promote polarization toward a pro-inflammatory M1-like phenotype, and suppress polarization toward an anti-inflammatory, reparative M2-like phenotype, thereby disrupting systemic immune homeostasis (Cifarelli et al., 2020; Huang et al., 2024). This imbalance in macrophage polarization, mediated by BCAA metabolic dysfunction, leads to the sustained release of large amounts of pro-inflammatory factors, amplifying local and systemic low-grade chronic inflammation. This further exacerbates IR and impairs pancreatic β-cell function, ultimately driving the onset and progression of diabetes and its complications.

Based on this, we propose that BCAA metabolic dysregulation may drive an imbalance in macrophage polarization by modulating immunometabolic reprogramming, thereby contributing to the onset and progression of metabolic inflammation in diabetes. However, the current body of evidence linking BCAA metabolism, immunometabolic reprogramming, macrophage polarization, and diabetes remains incomplete. Existing studies have largely focused on relatively independent, single-pathway mechanisms, such as BCAA metabolism and diabetes onset, macrophage polarization and metabolic inflammation in diabetes, as well as BCAA-mediated macrophage polarization. However, they lack a systematic integration of the intrinsic connections among these components. Based on the current state of research, this paper uses PubMed, Web of Science, and Google Scholar as search databases to collect relevant literature published from the inception of these databases through May 2026. Focusing on the interdisciplinary fields of BCAA metabolism, immunometabolic reprogramming, macrophage polarization, and metabolic inflammation in diabetes, this study systematically examines the molecular regulatory mechanisms underlying BCAA catabolic dysregulation in the pathological state of diabetes. This study provides the first systematic summary of the complete molecular mechanism by which BCAA catabolic dysregulation regulates macrophage polarization through immunometabolic reprogramming. Additionally, it moves beyond previous studies focused on single intervention methods to systematically summarize intervention strategies that target BCAA metabolism to regulate macrophage polarization and metabolic inflammation in diabetes. The aim is to provide new research insights for interventions targeting metabolic inflammation in diabetes by regulating BCAA metabolism.

## 2 Characteristics and molecular mechanisms of BCAA metabolic dysregulation in diabetes

### 2.1 Physiological metabolic pathways of BCAA

BCAA is an essential amino acid for the human body; it serves not only as a substrate for protein synthesis but also as an important regulator of nutrient sensing, energy metabolism, and cellular signal transduction (Neinast et al., 2019). The catabolism of BCAA primarily involves two initial steps. The first step is transamination, in which BCAA, catalyzed by branched-chain aminotransferase (BCAT), typically transfer their amino groups to α-KG, which acts as the amino acceptor to form glutamate and the corresponding branched-chain α-keto acids (BCKAs) (Hutson et al., 2005). The second step is an irreversible oxidative decarboxylation reaction, catalyzed by the branched-chain α-keto acid dehydrogenase complex (BCKDH/BCKD) in the mitochondria. This reaction converts the three BCKAs into their corresponding branched-chain acyl-CoA derivatives, releases CO_2_, and generates reducing equivalents (Reifenberg and Zimmer, 2024). The activity of the BCKDH/BCKD complex is regulated by phosphorylation: BCKDK phosphorylates BCKDHA (the E1α subunit of the BCKDH/BCKD complex), thereby inhibiting BCKDH/BCKD activity, while the phosphatase PPM1K, also known as PP2Cm, can dephosphorylate and activate the BCKDH/BCKD complex (White et al., 2018). Research suggests that BCAA catabolism exhibits marked tissue specificity (Mu et al., 2023). Unlike most amino acids, which undergo primarily hepatic first-pass metabolism, BCAAs undergo relatively little hepatic first-pass metabolism, and peripheral tissues such as skeletal muscle, adipose tissue, heart, and brown adipose tissue make significant contributions to BCAA clearance (Holeček, 2018). Herman et al. (2010) demonstrated that adipose tissue has the ability to regulate circulating BCAA levels, and that downregulation of BCAA oxidase expression in adipose tissue under conditions of obesity and IR may affect circulating BCAA levels. SLC25A44 is a mitochondrial BCAA transporter identified in recent years. Yoneshiro et al. (2019) found that brown adipose tissue can utilize BCAAs for mitochondrial catabolism and thermogenesis during cold exposure; SLC25A44 mediates the entry of BCAAs into mitochondria and plays a crucial role in BCAA clearance and the regulation of energy homeostasis in brown adipose tissue. Furthermore, in immune cells, BCAA metabolism also plays a role in regulating macrophage function. Studies have shown (Lu et al., 2024) that BCAA metabolism is one of the metabolic pathways significantly reprogrammed in IL-4-induced M2 macrophages; BCAA supplementation promotes M2 macrophage polarization and enhances oxidative phosphorylation levels, whereas knockdown of SLC25A44, BCAT2, or BCKDHA inhibits M2 macrophage polarization. These findings suggest that, under physiological conditions, BCAA catabolism provides important metabolic support for the oxidative metabolism and reparative functions of M2-like macrophages during alternative macrophage activation.

### 2.2 Pathological characteristics of BCAA metabolic dysregulation in diabetes

**Figure 1:**
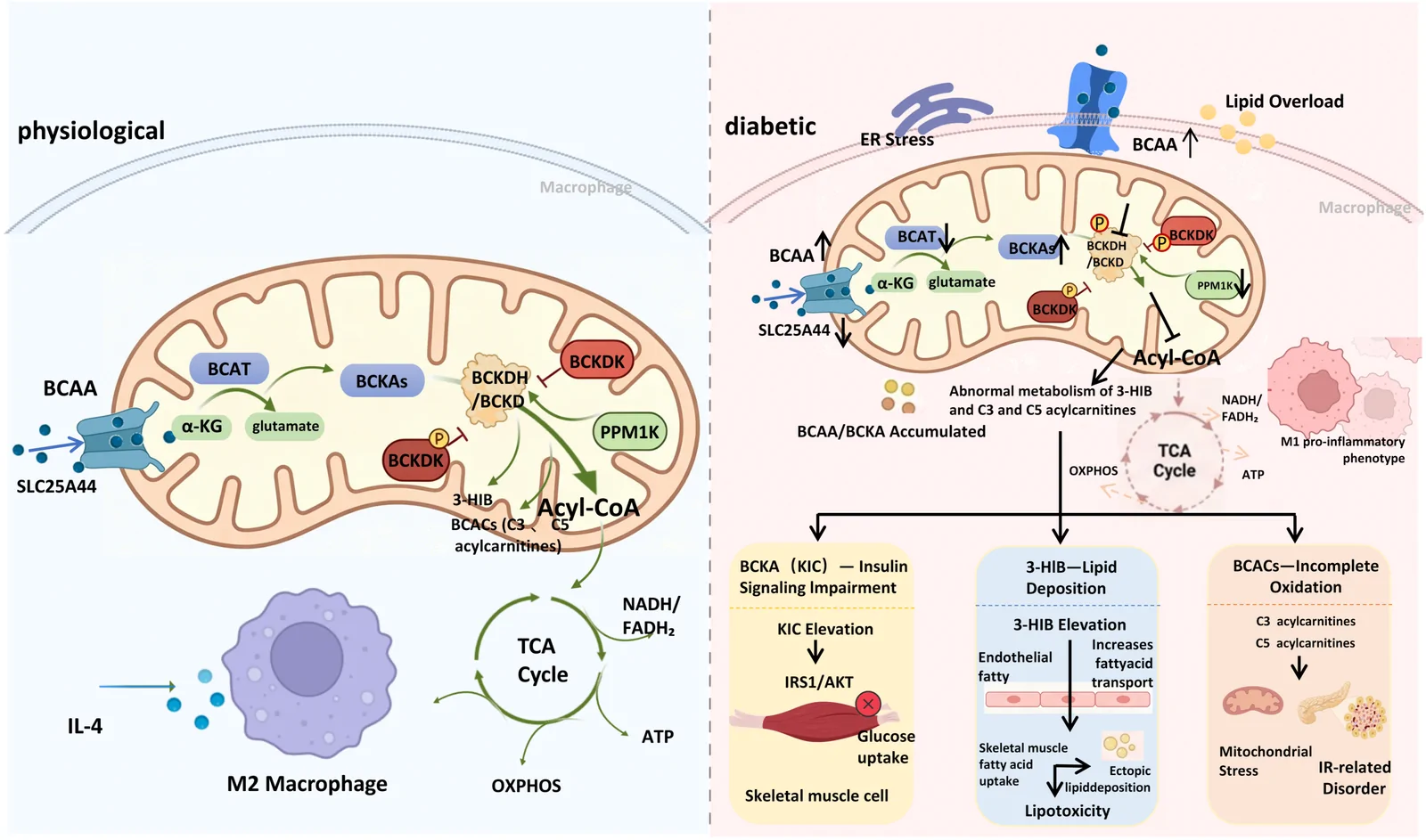
BCAA catabolism under physiological and diabetic conditions.

Numerous studies have shown that elevated circulating BCAA levels are one of the key metabolic hallmarks of IR and T2DM (Newgard et al., 2009; Flores-Guerrero et al., 2018; Rivas-Tumanyan et al., 2024). Zhao et al. (2016) conducted a systematic review including 23 studies and 20,091 participants and reported that BCAA may serve as useful biomarkers for IR and subsequent diabetes risk. A Mendelian randomization study found that genetically predicted impaired BCAA breakdown was associated with an increased risk of T2DM, suggesting that BCAA metabolic defects may play a causal role (Lotta et al., 2016). Another study indicated that genetically predicted IR can lead to elevated circulating BCAA levels (Mahendran et al., 2017). Furthermore, a Mendelian randomization study clearly demonstrated that IR directly affects BCAA metabolism and inflammatory responses. It suggests that BCAA metabolism is a link in the causal pathway from obesity and IR to T2DM (Wang et al., 2017). In diabetes or IR, the decreased BCAA catabolic flux is closely associated with reduced BCAT activity and inhibition of the BCKD/BCKDH complex (White et al., 2018; David et al., 2019). When PPM1K expression or activity is reduced, insufficient dephosphorylation of the BCKD/BCKDH complex leads to decreased oxidative decarboxylation activity, which impedes the conversion of BCKA to the corresponding branched-chain acyl-CoA, thereby promoting BCKA accumulation and disrupting BCAA catabolism (Lu et al., 2009; Nishi et al., 2023). Concurrently, incomplete BCAA downstream oxidation or increased mitochondrial metabolic stress may be accompanied by alterations in abnormal metabolites such as 3-HIB and C3/C5 branched-chain acylcarnitines (De Bandt et al., 2022). Biswas et al. (2020) found that BCAAs and their ketoacid derivatives, particularly α-ketoisocaproate (KIC), can inhibit insulin-stimulated glucose uptake in muscle cells and impair IRS1/AKT signaling. C3 and C5 acylcarnitines are common branched-chain acylcarnitines (BCACs) produced by BCAA catabolism. Elevated levels of C3 and C5 acylcarnitines indicate increased BCAA catabolic flux or insufficient downstream mitochondrial oxidative capacity, leading to the accumulation of BCAA-derived acyl-CoA intermediates in the form of acylcarnitines (Newgard et al., 2009; Arany and Neinast, 2018). Batch et al. (2013) found that C3 and C5 acylcarnitines cluster with BCAAs to form a metabolic signature associated with IR. 3-HIB is a metabolite of valine. Studies have shown that it is secreted by muscle cells, promotes endothelial fatty acid transport, increases fatty acid uptake and lipid accumulation in skeletal muscle, and induces IR (Jang et al., 2016; Nilsen et al., 2020). The reduced ability to metabolize BCAAs in skeletal muscle, liver, and adipose tissue is considered one of the key pathophysiological features of IR and T2DM. Burrill et al. (2015) found that inflammation and endoplasmic reticulum stress can downregulate the expression of BCAA metabolism-related genes in adipocytes, suggesting that the inflammatory microenvironment associated with obesity can directly impair BCAA metabolism in adipose tissue. Research by Blair et al. (2023) indicates that BCAA catabolism in skeletal muscle can influence systemic BCAA levels; however, reducing fasting plasma BCAA levels alone is insufficient to improve insulin sensitivity. Shin et al. (2014) demonstrated that insulin action in the brain reduces circulating BCAA and induces hepatic BCKDH expression and activity, whereas IR in obesity and prediabetes impairs this regulation. Current evidence suggests that BCAA metabolism plays a context-dependent role in macrophage polarization. Under physiological conditions, BCAA catabolism plays an important role in supporting macrophage polarization toward an M2-like phenotype. Under pathological conditions such as diabetes and obesity, however, chronic BCAA accumulation and impaired BCAA catabolism may lead to BCKA overload, including KIC accumulation, increased 3-HIB levels, and incomplete oxidation of BCAA-derived metabolites, thereby promoting pro-inflammatory responses through multiple pathways. The characteristics of BCAA catabolism under physiological and diabetic conditions are shown in **Figure 1**.

### 2.3 Molecular mechanisms regulating BCAA metabolic abnormalities in diabetes

Mechanistic target of rapamycin complex 1 (mTORC1) serves as a central signaling hub that senses BCAA availability and coordinates cellular metabolism, while its dysregulation contributes to metabolic abnormalities. BCAA, particularly leucine, can activate the mTORC1 signaling pathway, while chronic overnutrition and sustained mTORC1/S6K1 activation contribute to IR by inhibiting insulin receptor substrate signaling (Yoon, 2016). Studies have explicitly demonstrated that BCAA-induced activation of mTORC1 is a key mechanism linking BCAA to IR. In a study of mouse hearts, Zhen et al. (2016) found that rapamycin-mediated inhibition of mTORC1 significantly suppressed leucine-induced BCKDH activation, suggesting that mTORC1 participates in the regulation of the BCKDH complex, the rate-limiting enzyme in BCAA catabolism. Furthermore, the study suggests that chronic inflammation, lipid overload, and endoplasmic reticulum stress in states of obesity and diabetes can further suppress the expression of genes and enzymes related to BCAA catabolism, such as BCAT, BCKDHA, BCKDHB (the β subunit of the E1 component of the BCKDH complex), DBT (the E2 transacylase subunit of the BCKDH complex), DLD (the E3 dihydrolipoamide dehydrogenase subunit of the BCKDH complex), and PPM1K, thereby reducing BCAA oxidative catabolism capacity and promoting the continuous accumulation of BCAA/BCKA and their derivative metabolites (Burrill et al., 2015). Concurrently, 3-HIB, a byproduct of abnormal BCAA metabolism, can exacerbate lipid mislocalization and lipotoxic damage in skeletal muscle by promoting endothelial fatty acid transport. By enhancing endothelial fatty acid transport and increasing lipid uptake in skeletal muscle, 3-HIB may provide a mechanistic link between BCAA metabolic dysregulation, lipid overload, and skeletal muscle IR (Jang et al., 2016). Furthermore, signal transducer and activator of transcription 1 (STAT1), as a key transcription factor, enters the nucleus following phosphorylation by receptor-associated kinases to regulate the expression of immune-related genes (Hu et al., 2021). Studies have shown that STAT1 acts as a “transcriptional hub for pro-inflammatory polarization” during abnormal BCAA metabolism. This is because elevated BCAA levels lead to increased phosphorylation of interferon gamma receptor 1 (IFNGR1), janus kinase 1 (JAK1), and STAT1 in adipose tissue macrophages, accompanied by elevated iNOS and M1-like polarization (Huang et al., 2024). NF-κB is a key transcriptional regulator of inflammatory signal transduction, cytokine release, inflammasome activation, and M1-like polarization in monocytes/macrophages (Mussbacher et al., 2023). Studies have shown that it primarily amplifies inflammatory effects during BCAA catabolism. High concentrations of BCAAs can promote the release of inflammatory molecules such as IL-6 and TNF-α in peripheral blood mononuclear cells through the activation of AKT-mTORC1, reactive oxygen species (ROS), and NF-κB (Zhenyukh et al., 2017). Therefore, the molecular basis of BCAA metabolic abnormalities in diabetes primarily includes reduced expression or activity of BCAA-degrading enzymes, abnormal phosphorylation regulation of the BCKDH/BCKD complex, suppression of BCAA-degrading genes by inflammation and endoplasmic reticulum stress, mitochondrial metabolic dysfunction, and excessive activation of pro-inflammatory signaling pathways such as mTORC1, STAT1, and NF-κB.

**Table 1** summarizes the key molecules involved in BCAA metabolism and their functional relevance to macrophage polarization and diabetic metabolic inflammation.

**Table 1:** Key molecules involved in BCAA metabolism and their functional relevance to macrophage polarization and diabetic metabolic inflammation.

Molecule or node	Category	Function in BCAA metabolism	Relevance to macrophage polarization or diabetic inflammation	References
BCAT (BCAT1/BCAT2)	Enzyme	Catalyzes transamination of BCAAs to BCKAs	BCAT2 knockdown inhibits M2 macrophage polarization; involved in altered BCAA catabolism in obesity/IR	(Hutson et al., 2005; Burrill et al., 2015; Lu et al., 2024)
BCKDH / BCKD complex (BCKDHA/BCKDHB/DBT/DLD)	Enzyme complex	Irreversible oxidative decarboxylation of BCKAs to acyl-CoAs	Impaired activity leads to BCAA accumulation, contributes to IR and metabolic inflammation	(Lu et al., 2009; Shin et al., 2014; Burrill et al., 2015; Zhen et al., 2016; White et al., 2018; Nishi et al., 2023; Reifenberg and Zimmer, 2024)
BCKAs	Metabolites	Products of BCAA transamination	Accumulation reflects impaired BCAA catabolism and may worsen IR	(Hutson et al., 2005; Burrill et al., 2015; Nishi et al., 2023)
α-KG	Amino-group acceptor / TCA intermediate	Accepts amino groups from BCAAs	Links BCAA transamination with TCA metabolism; regulation of macrophage activation	(Hutson et al., 2005)
KIC	Leucine-derived BCKA	Leucine-derived keto acid	Inhibits insulin-stimulated glucose uptake and IRS1/AKT signaling	(Biswas et al., 2020)
Glutamate	Product of transamination	Generated from α-KG during transamination	Reflects BCAA transamination	(Hutson et al., 2005)
BCKDK	Kinase	Phosphorylates and inhibits BCKDH	Overactivation suppresses BCAA catabolism, exacerbating IR	(White et al., 2018)
PPM1K (PP2Cm)	Phosphatase	Dephosphorylates and activates BCKDH	Deficiency impairs BCAA catabolism; linked to IR and diabetic metabolic dysfunction	(Lu et al., 2009; Burrill et al., 2015; White et al., 2018; Nishi et al., 2023)
SLC25A44	Mitochondrial transporter	Mediates BCAA transport into mitochondria	Essential for M2 macrophage polarization and BCAA catabolism in brown adipose tissue	(Yoneshiro et al., 2019; Lu et al., 2024)
mTORC1	Signaling hub	Senses leucine/BCAA levels; regulates BCAA catabolism	Chronic activation contributes to IR via inhibition of insulin signaling; mediates inflammation	(Yoon, 2016; Zhen et al., 2016)
STAT1	Transcription factor	Regulates immune-related gene expression	Acts as hub for pro-inflammatory M1 polarization under elevated BCAA conditions	(Hu et al., 2021; Huang et al., 2024)
NF-κB	Transcription factor	Regulates inflammatory cytokines	Amplifies inflammatory response during BCAA dysregulation; promotes M1 polarization	(Zhenyukh et al., 2017; Mussbacher et al., 2023)
3-HIB	Metabolite (valine-derived)	Intermediate of valine catabolism	Promotes endothelial FA transport, lipid accumulation in muscle, induces insulin resistance	(Jang et al., 2016; Nilsen et al., 2020; De Bandt et al., 2022)
C3 and C5 acylcarnitines	Metabolite	Reflect BCAA catabolism / incomplete oxidation	Associated with IR	(Newgard et al., 2009; Batch et al., 2013; Arany and Neinast, 2018; De Bandt et al., 2022)

## 3 mechanistic study of how BCAA metabolic dysregulation mediates metabolic reprogramming and polarization imbalance in macrophages

BCAAs not only serve as important substrates for protein synthesis and energy metabolism but also participate in the metabolic reprogramming and functional activation of macrophages. Current research suggests that the effects of BCAAs on macrophage phenotype and function are highly tissue- and stimulus-dependent. At the tissue level, mononuclear macrophages infiltrating adipose tissue are most sensitive to BCAA accumulation; pathological BCAA accumulation can strongly drive M1 polarization and suppress the M2 reparative phenotype (Huang et al., 2024); in the liver, BCAA exposure alone only slightly alters the macrophage phenotype, but when co-stimulated with endotoxin (LPS), BCAA accumulation can significantly amplify the pro-inflammatory transcriptional program in hepatic macrophages (Doshi et al., 2026); intestinal mucosal macrophages, in turn, regulate intestinal inflammatory homeostasis through the synergistic interaction between the microbiota and BCAA (Chen et al., 2025). During skeletal muscle injury repair, BCAA first enhances the function of inflammatory macrophages during the damage clearance and cell proliferation phases, and subsequently enhance the function of reparative macrophages during the phases of inflammation resolution, myocyte differentiation, and tissue remodeling (Dong et al., 2022). From a stimulus-dependent perspective, BCAAs do not exert uniformly pro-inflammatory or anti-inflammatory effects; rather, they act as nutrient-sensing signals and metabolic substrates that modulate pre-existing macrophage activation programs. In LPS-induced pro-inflammatory activation, BCAA can enhance the mTORC1–HIF-1α–glycolysis signaling pathway, further increasing the expression of M1-associated genes (Dong et al., 2022); in the context of BCAA accumulation, the IFNGR1/JAK1/STAT1 pathway also contributes to the pro-inflammatory shift in adipose tissue macrophages (Huang et al., 2024).In contrast, during IL-4-driven alternative activation, mitochondrial BCAA transport and the degradation processes mediated by BCAT2 and BCKDHA support oxidative metabolism and the expression of M2-associated genes (Lu et al., 2024). Thus, BCAA dysregulation serves as a condition-dependent metabolic switch regulating macrophage polarization, with tissue-specific metabolic characteristics and the type of exogenous stimulus jointly determining whether the final outcome leans toward pro-inflammatory M1 or anti-inflammatory M2 repair. Based on this, the present study summarizes the mechanisms by which BCAA metabolic dysregulation mediates macrophage immunometabolic reprogramming and polarization imbalance as follows:

### 3.1 The BCAA-mTORC1-HIF-1α-glycolysis axis promotes M1-like pro-inflammatory metabolic reprogramming

BCAAs are key upstream signals in the mTORC1 nutrient-sensing pathway, with leucine playing a particularly prominent role in activating mTORC1 (Dimou et al., 2022). In macrophages, leucine uptake mediated by SLC7A5 (a large neutral amino acid transporter located on the cell membrane) has been shown to induce glycolytic reprogramming via mTORC1 and promote the production of pro-inflammatory factors (Kandasamy et al., 2018). In a study of human macrophages, Yoon et al. (2018) found that LPS stimulation induced SLC7A5 expression; whereas pharmacological inhibition or silencing of SLC7A5 reduced leucine-mediated mTORC1 activation, lowered the extracellular acidification rate, and significantly decreased IL-1β production. This study provides direct experimental evidence that leucine activates mTORC1, induces glycolytic reprogramming, and promotes M1-like pro-inflammatory metabolism. Hypoxia-inducible factor-1 alpha (HIF-1α), a central transcriptional regulator in cellular adaptation to hypoxia and metabolic stress, promotes glycolytic metabolism and enhances M1-like pro-inflammatory function (Cramer et al., 2003). Further studies have indicated that mTORC1, as a nutrient-sensing complex, can induce metabolic reprogramming in an HIF-1α-dependent manner. Activation of mTORC1 upregulates the expression of genes involved in glycolysis, thereby driving cells toward a glycolytic phenotype and promoting the expression of glycolytic enzymes and pro-inflammatory mediators (Düvel et al., 2010; Cheng et al., 2014). Huang et al. (2024) found in mice fed a high-BCAA diet that 16 weeks of BCAA supplementation increased levels of IL-1β, TNF-α, and MCP-1 in adipose tissue; simultaneously, the proportion of CD206^+^ cells decreased while the proportion of CD86^+^ cells increased in adipose tissue macrophages, suggesting that BCAA supplementation promoted a shift in adipose tissue macrophages from an anti-inflammatory to a pro-inflammatory phenotype. The study also demonstrated enhanced iNOS signaling in adipose tissue macrophages in the high-BCAA group, further supporting their M1-like pro-inflammatory polarization. Therefore, BCAAs, particularly leucine, can act as nutritional signals that enter macrophages via SLC7A5 and promote glycolytic reprogramming and the production of inflammatory factors through mTORC1; the mTORC1-HIF-1α-glycolysis pathway is a key mechanism underlying M1-like pro-inflammatory metabolic reprogramming.

### 3.2 Impaired BCAA catabolism disrupts the TCA cycle and OXPHOS, suppressing the M2-like repair phenotype

The carbon skeletons generated during BCAA catabolism can be converted into acetyl-CoA, acetoacetate, or succinyl-CoA. These metabolites contribute to the TCA cycle either directly or after further conversion, thereby generating reducing equivalents in the form of nicotinamide adenine dinucleotide (NADH) and flavin adenine dinucleotide (FADH_2_) to support mitochondrial OXPHOS and ATP synthesis (Huang et al., 2014; Kieler et al., 2021). M2-like macrophages possess a relatively intact TCA cycle and strong OXPHOS characteristics. Studies indicate that M1 activation inhibits mitochondrial OXPHOS in macrophages and impedes their repolarization to the M2 phenotype; restoring mitochondrial function facilitates the reprogramming of pro-inflammatory macrophages to an anti-inflammatory phenotype (Corcoran and O’Neill, 2016). Direct experimental evidence supports the involvement of BCAA catabolism in M2 polarization. Lu et al. (2024) found that BCAAs are transported into mitochondria by SLC25A44 and subsequently transaminated by BCAT2 to generate branched-chain α-keto acids (BCKAs). The resulting BCKAs undergo oxidative decarboxylation by the BCKDH complex, ultimately contributing to the production of acetyl-CoA and succinyl-CoA. These metabolites support TCA cycle activity and promote the generation of NADH and FADH_2_ to fuel OXPHOS. In parallel, acetyl-CoA serves as a substrate for histone acetylation, thereby promoting the transcription of M2-associated genes in bone marrow-derived macrophages. When the expression of key molecules involved in BCAA breakdown (SLC25A44, BCAT2, BCKDHA) is downregulated or their function is impaired, mitochondrial BCAA substrate supply is insufficient, leading to reduced production of acetyl-CoA and succinyl-CoA. This directly reduces TCA cycle flux, suppresses OXPHOS and ATP synthesis. In parallel, reduced acetyl-CoA availability decreases histone acetylation, thereby repressing the transcription of M2-associated anti-inflammatory and reparative genes, including Arg1 and Mrc1, the latter of which encodes CD206, and ultimately inhibiting M2-like macrophage polarization (Lu et al., 2024). Therefore, the role of BCAA catabolism in supporting the M2-like reparative phenotype depends on mitochondrial transport and oxidative degradation processes; knockdown of SLC25A44, BCAT2, and BCKDHA inhibits M2-like macrophage polarization. Impaired BCAA catabolism in diabetic or obese states disrupts the mitochondrial metabolic foundation required for M2 polarization by impairing TCA cycle replenishment and OXPHOS capacity.

### 3.3 BCKA, 3-HIB, and BCAC-mediated lipotoxic inflammation and macrophage activation

BCKAs are the direct products of BCAA transamination catalyzed by BCAT enzymes, and their subsequent oxidative decarboxylation depends on the BCKDH complex. Liu et al. (2021) found that BCKDH activity was reduced in multiple organs of db/db diabetic mice, thereby impairing BCKA oxidation and clearance. Long-term accumulation of BCKA significantly exacerbates inflammation and organ damage. This was evidenced by elevated levels of MCP-1 and IL-6 in serum; upregulated mRNA expression of IL-1β, IL-6, and MCP-1 in liver, kidney, and skeletal muscle tissues; increased protein expression of IL-1β and IL-6 in liver and kidney tissues; and increased infiltration of F4/80^+^ macrophages in liver and kidney tissues. This study suggests that BCKA accumulation exacerbates macrophage inflammatory activation, oxidative stress, and chronic liver and kidney damage in the context of diabetes. 3-HIB can be secreted by myocytes and act as a paracrine signal that promotes endothelial fatty acid transport, thereby increasing fatty acid uptake by skeletal muscle and intramuscular lipid accumulation. Jang et al. (2016) found that 3-HIB induces the accumulation of triacylglycerols and diacylglycerols, promotes PKC-θ membrane translocation, and inhibits AKT phosphorylation, ultimately leading to impaired glucose tolerance and lipotoxic IR. Therefore, 3-HIB may serve as a key metabolic mediator linking BCAA metabolic abnormalities, lipotoxic damage, and the inflammatory microenvironment of diabetes, providing a pathological basis for macrophage recruitment and pro-inflammatory activation. BCACs are common metabolic markers of incomplete BCAA and fatty acid oxidation. In rat experiments, Newgard et al. (2009) found that a high-fat diet supplemented with BCAAs led to the accumulation of C3 and C5 acylcarnitines and other acylcarnitine species in skeletal muscle. These metabolic alterations were accompanied by activation of mTOR/S6K1 and JNK signaling, increased serine phosphorylation of IRS1, and impaired insulin-stimulated AKT phosphorylation. Importantly, rapamycin ameliorated insulin resistance and attenuated the inflammatory response in this model.

### 3.4 The BCAA-mitochondrial ROS-endoplasmic reticulum stress-NLRP3 inflammasome axis amplifies pro-inflammatory responses

There is a close link between mitochondrial ROS and pro-inflammatory activation of macrophages. In an atherosclerosis model, Zhao et al. (2023) found that elevated BCAA levels promoted the pro-inflammatory activation of macrophages. Specifically, excess BCAAs induced mitochondria-to-nucleus H_2_O_2_ signaling, which promoted the formation and secretion of disulfide HMGB1 and thereby triggered an inflammatory cascade in mouse peritoneal macrophages. However, scavenging H_2_O_2_ with a nuclear-targeted peroxidase inhibited BCAA-induced macrophage inflammation. The NLRP3 inflammasome is a key inflammatory amplifier in the chronic inflammation associated with obesity, IR, and T2DM (Vandanmagsar et al., 2011). Litwiniuk et al. (2021) noted that obesity-related inflammatory factors promote inflammasome complex formation and lead to the maturation and release of pro-inflammatory cytokines such as IL-1β and IL-18. Lv et al. (2021) further found that endoplasmic reticulum stress (ERS) activated the NLRP3 inflammasome by increasing mitochondrial ROS production, disrupting calcium homeostasis, and activating the unfolded protein response (UPR), thereby promoting the maturation of IL-1β and IL-18. This represents a key mechanism underlying the development of chronic inflammation and IR in diabetes. Therefore, we hypothesize that BCAA accumulation induces excessive mitochondrial ROS production, which in turn triggers endoplasmic reticulum stress. This, through calcium homeostasis disruption and UPR signaling, activates the NLRP3 inflammasome, amplifies the release of pro-inflammatory factors, and collectively drives chronic inflammation and IR in diabetes.

### 3.5 BCAA mediates inflammatory pathways such as IFNGR1/JAK1/STAT1 and NF-κB to maintain M1-like polarization

The IFNGR1/JAK1/STAT1 signaling axis is a key component of IFN-γ-mediated classical macrophage activation. Upon IFN-γ binding to the IFN-γ receptor complex, receptor-associated JAK kinases are activated and phosphorylate the receptor subunits, thereby creating docking sites for STAT1. STAT1 is subsequently phosphorylated, dimerizes, and translocates to the nucleus, where it induces the transcription of IFN-γ-responsive genes. IFN-γ can induce macrophage activation toward the M1 phenotype in a STAT1-dependent manner and promote inflammatory immune responses; the IFN-γ/STAT1 pathway also regulates Toll-like receptor (TLR) signaling, inflammatory factors, and cytokine responses associated with tissue damage (Schroder et al., 2004; Chen et al., 2023). In a study investigating the effects of high BCAA levels on the pro-inflammatory polarization of macrophages, the results showed that high BCAA supplementation increased IFNGR1 protein expression in adipose tissue macrophages but did not increase IFNGR2 expression. Concurrently, high-BCAA supplementation increased the p-JAK1/JAK1 and p-STAT1/STAT1 ratios and elevated iNOS expression, indicating activation of the IFNGR1/JAK1/STAT1 pathway and macrophage polarization toward a pro-inflammatory phenotype. Furthermore, the study validated through IFNGR1 silencing experiments that IFNGR1 is a key target for high-BCAA stimulation-induced inflammation in adipose tissue and pro-inflammatory macrophage polarization (Huang et al., 2024). Nuclear factor-κB (NF-κB) is a central transcription factor in the inflammatory response of macrophages. Pathogen-associated molecular patterns (PAMPs) and damage-associated molecular patterns (DAMPs) activate TLRs and other pattern-recognition receptors, leading to the recruitment of adaptor proteins such as myeloid differentiation primary response protein 88 (MyD88) and TIR-domain-containing adapter-inducing interferon-β (TRIF). These signaling events activate transforming growth factor-β-activated kinase 1 (TAK1) and the IκB kinase (IKK) complex, resulting in the phosphorylation and proteasomal degradation of inhibitor of NF-κB α (IκBα). This allows NF-κB to enter the cell nucleus and induce the expression of inflammatory genes (Lawrence, 2009). In the aforementioned study, RNA-seq and GSEA data revealed that TNF-α signaling via the NF-κB pathway was significantly enriched in macrophages following high BCAA supplementation; protein expression of IL-1β, TNF-α, and MCP-1 was significantly elevated in adipose tissue, and the proportion of CD86^+^ pro-inflammatory macrophages increased. Therefore, the study suggests that under conditions of high BCAA levels, the transcriptional profiles of macrophages exhibit an enrichment of the pro-inflammatory IFN-γ/JAK/STAT and TNF-α/NF-κB pathways, accompanied by an enhanced M1-like phenotype. Tang et al. (2026) also found that systemic BCAA accumulation activated the mTORC1–NF-κB inflammatory signaling cascade, upregulated pro-inflammatory mediators such as IL-1β, TNF-α, and MCP-1 in macrophages, and drove macrophages toward a CD86^+^/iNOS^+^ M1-like pro-inflammatory phenotype.

**Figure 2:**
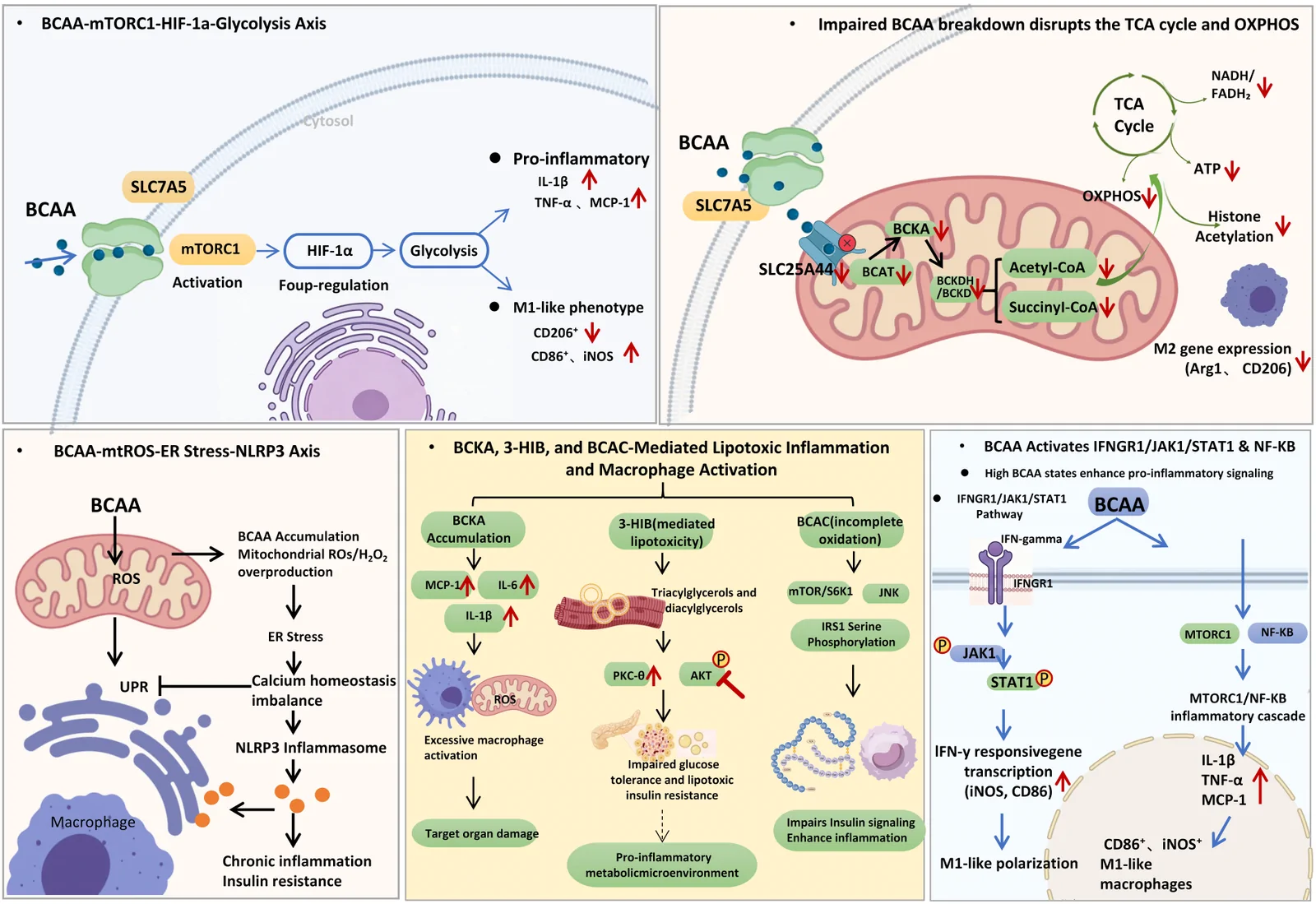
BCAA metabolic dysregulation mediates macrophage metabolic reprogramming and polarization imbalance.

See **Figure 2** for a schematic diagram illustrating the overall mechanism by which BCAA metabolic dysregulation mediates the metabolic reprogramming and polarization imbalance of macrophages.

## 4 Intervention strategies targeting BCAA metabolism to regulate macrophage polarization and diabetic metabolic inflammation

### 4.1 Low-BCAA diets and calorie restriction

A low-BCAA diet is currently one of the most direct nutritional interventions; its core mechanism involves reducing the exogenous BCAA load, thereby improving metabolic disorders associated with obesity and IR (Cummings et al., 2018). Based on evidence linking elevated BCAA levels and T2DM, Fontana et al. (2016) conducted calorie-controlled experiments in mice and a randomized controlled trial in humans. Their results demonstrated that a diet specifically restricted in BCAAs significantly improved glucose tolerance and enhanced insulin sensitivity and that these effects were independent of calorie restriction. Building on their previous findings that dietary BCAA restriction reduced adipose tissue accumulation and adipocyte hypertrophy and improved insulin resistance, Liu et al. (2022) conducted a detailed mechanistic investigation. The study showed that a high-fat diet reduced the abundance of M2-like macrophages in adipose tissue, whereas dietary BCAA restriction prevented this decline, suggesting that limiting BCAA intake may preserve M2-like macrophage polarization under conditions of metabolic inflammation. Zhang et al. (2025b) also found that in mice with high-fat diet-induced obesity, long-term dietary BCAA restriction slightly improved blood glucose and lipid levels and reduced macrophage infiltration in adipose tissue along with the expression of pro-inflammatory factors (TNF-α, IL-1β, IL-6); however, the beneficial effects of this intervention were limited and not statistically significant.

### 4.2 Exercise Intervention

Existing research indicates that the beneficial effects of exercise on BCAA metabolism are primarily reflected in the restoration of intracellular catabolic flux and the upregulation of metabolic enzymes. Glynn et al. (2015) found that six months of combined aerobic and resistance training in overweight, sedentary participants with IR improved insulin sensitivity and enhanced systemic leucine turnover and BCAA metabolic capacity. Lee et al. (2021) further found that exercise upregulated BCAA metabolic pathways in skeletal muscle and subcutaneous white adipose tissue, as evidenced by increased expression of BCKDH-related genes and the mitochondrial BCAA transporter SLC25A44, together with reduced BCKDK expression in adipose tissue in participants with impaired glucose metabolism. In mice on a high-fat diet, 8 weeks of continuous aerobic exercise suppressed the accumulation of M1-like macrophages in skeletal muscle, reduced IL-1β and iNOS levels, increased IL-10 and M2-related signaling, and improved skeletal muscle insulin resistance. Further studies have shown that exercise downregulates miR-221-3p in skeletal muscle, thereby relieving miR-221-3p-mediated suppression of SOCS1 and consequently attenuating pro-inflammatory JAK/STAT signaling and STAT1-mediated M1 macrophage polarization (Li et al., 2022, 2025). This evidence is mechanistically consistent with studies showing that BCAA accumulation promotes pro-inflammatory macrophage polarization through the IFNGR1/JAK1/STAT1 pathway (Huang et al., 2024), suggesting that JAK/STAT signaling may represent a shared mechanistic target linking BCAA metabolic abnormalities to the anti-inflammatory effects of exercise. We therefore hypothesize that exercise therapy, on the one hand, increases the expression of BCAA oxidation-related enzymes and mitochondrial transporters in skeletal muscle and white adipose tissue, thereby enhancing the tissue clearance of BCAAs and BCKAs and their utilization in the tricarboxylic acid cycle; on the other hand, it may reduce lipotoxicity, tissue hypoxia, and inflammatory stimulation, inhibit the IFNGR1/JAK1/STAT1 pathway and related pro-inflammatory signaling, and provide a more favorable metabolic environment for M2-like macrophages that rely on oxidative phosphorylation, thereby alleviating the metabolic inflammatory response associated with diabetes.

### 4.3 BCAA catabolic activator: BT2

BT2 is a widely used BCKDK inhibitor that prevents BCKDK-mediated phosphorylation and the consequent inactivation of BCKDH, thereby increasing BCKDH activity and enhancing oxidative flux through BCAA and BCKA catabolism (Tso et al., 2014). Research by Zhou et al. (2019a) demonstrated that BT2 can increase BCKD activity, lower plasma BCAA and BCKA levels, and inhibit the phosphorylation of the BCKD E1α protein in the liver; by inhibiting mTORC1 activity, it improves insulin sensitivity in diet-induced obese mice, providing strong experimental evidence for the use of BT2 in treating IR. Bollinger et al. (2022) further demonstrated that BT2 significantly enhanced BCAA catabolism, reduced BCAA and BCKA levels in plasma and tissues, improved insulin sensitivity and glucose tolerance in diet-induced obese mice, and alleviated hepatic steatosis and liver injury. The specific mechanism involves BT2 systemically reducing BCAA/BCKA levels, thereby inhibiting M1 pro-inflammatory polarization of macrophages and reducing the infiltration of ionized calcium-binding adapter molecule 1-positive (Iba1^+^) macrophages in the liver. Additionally, it suppresses the release of pro-inflammatory factors (TNF-α, C-C motif chemokine ligand 2 (CCL2)) while activating the hepatic PPARα pathway to promote fatty acid oxidation and drive macrophage shift toward the M2 anti-inflammatory and reparative phenotype.

### 4.4 PPARγ agonist: rosiglitazone

Rosiglitazone is a PPARγ agonist. Studies have shown that it reduces serum BCAA levels and upregulates BCAT and BCKDH activity in inguinal white adipose tissue and brown adipose tissue (Blanchard et al., 2018). PPARγ is a key transcriptional regulator of macrophage alternative activation. Studies by Odegaard et al. (2007) showed that PPARγ deficiency in myeloid cells impaired alternative macrophage activation and exacerbated diet-induced obesity and IR. A study by Rohm et al. (2024) further demonstrated that rosiglitazone significantly inhibited pro-inflammatory M1 polarization and promoted macrophage polarization toward the anti-inflammatory M2 phenotype by activating PPARγ in adipose tissue macrophages, thereby ameliorating the pro-inflammatory microenvironment. Specifically, rosiglitazone-treated macrophages secrete small extracellular vesicles (sEVs) rich in microRNAs. These sEVs circulated to insulin-responsive tissues, including adipose tissue, skeletal muscle, and the liver, and ameliorated glucose intolerance and insulin resistance in obese mice. Collectively, these findings suggest that rosiglitazone exerts beneficial metabolic effects through two complementary mechanisms. It can both regulate BCAA catabolism and promote the transition of adipose tissue macrophages to an anti-inflammatory and reparative state via PPARγ.

### 4.5 Berberine

Berberine is an isoquinoline alkaloid found in medicinal plants such as Coptis chinensis, Phellodendron amurense, and Coptis trifoliata, and is also one of the primary active components of Coptis chinensis (Neag et al., 2018). It exerts a broad range of pharmacological effects, including antidiarrheal, metabolism-regulating, cardioprotective, antibacterial, anti-inflammatory, and immunomodulatory effects (Song et al., 2020; Wang et al., 2024a). Consequently, BBR has emerged as an ideal candidate drug for intervening in BCAA metabolic disorders and regulating imbalances in macrophage polarization. Yue et al. (2019) demonstrated that BBR ameliorated glucose intolerance and IR in mice fed a high-fat diet, reduced inflammatory cell infiltration in the liver and adipose tissue, and lowered high-fat diet-induced elevations in serum BCAA levels. The specific mechanism involves berberine altering the composition of the gut microbiota, reducing the number of BCAA-producing bacteria, and promoting BCAA catabolism in the liver and epididymal white adipose tissue by decreasing BCKDHA phosphorylation and BCKDK levels. Jeong et al. (2009) found that, in macrophages isolated from db/db mice, BBR inhibited the phosphorylation of MAPKs, including p38, ERK, and JNK, reduced ROS levels, and significantly downregulated the expression of pro-inflammatory mediators, including TNF-α, IL-1β, IL-6, MCP-1, iNOS, and cyclooxygenase-2. Liu et al. (2018) further demonstrated that berberine inhibits pro-inflammatory M1 macrophage polarization in the colon via the AKT1/SOCS1/NF-κB signaling pathway. Collectively, these findings provide experimental support for the potential of berberine to modulate BCAA metabolism and macrophage polarization.

### 4.6 Ginsenoside Rb1

Ginsenoside Rb1 is a dammarane-type triterpenoid saponin and a major bioactive constituent of Araliaceae plants, particularly Korean ginseng (*Panax ginseng*) and American ginseng (*Panax quinquefolius*) (Hou et al., 2021). As a naturally occurring anti-inflammatory, hypoglycemic, and antioxidant molecule, ginsenoside Rb1 demonstrates potential therapeutic value in metabolic diseases such as diabetes, chronic inflammation, and fatty liver disease by regulating macrophage polarization and improving insulin signaling (Zhou et al., 2019b). Recent studies have further confirmed that ginsenoside Rb1 can also improve high-fat diet-induced IR by regulating gut microbiota and BCAA metabolism (Yang et al., 2021). Wang et al. (2024b) found that ginsenoside Rb1 significantly inhibited STING pathway activation in myocardial-infiltrating macrophages and suppressed the associated inflammatory response, thereby reducing pro-inflammatory cytokine release and alleviating macrophage-mediated inflammatory injury. Ding et al. (2023) also demonstrated that ginsenoside Rb1 inhibited the activation and migration of pro-inflammatory macrophages in adipose tissue, reduced inflammation, and ameliorated insulin resistance by upregulating PPARγ and inhibiting NF-κB signaling. Thus, ginsenoside Rb1 possesses the ability to regulate BCAA metabolism, improve IR, inhibit inflammatory macrophage activation, and promote anti-inflammatory polarization. These properties make ginsenoside Rb1 a promising single-compound candidate for modulating the BCAA–immune metabolic network.

### 4.7 Mulberry leaf/mulberry twig extracts

Extracts from mulberry leaves and twigs are rich in active compounds such as alkaloids, flavonoids, polysaccharides, and polyphenols, and have shown great potential in lowering blood sugar, reducing inflammation, regulating macrophage polarization, improving IR, and modulating BCAA metabolism (Cummings et al., 2018; Ma et al., 2022; Zheng et al., 2023). Studies have found that water extracts of mulberry leaves improve chronic inflammation and IR in mice with T2DM by inhibiting the TLR2/MyD88/NF-κB inflammatory pathway, reducing TNF-α, restoring insulin signaling (InsR/IRS1), and alleviating pancreatic inflammation (Tian et al., 2019). Sangzhi alkaloids (SZ-A), a natural alkaloid extract derived from mulberry twigs, exhibit a broad range of pharmacological activities and a favorable pharmacokinetic profile (Liu et al., 2023). Peng et al. (2024) found that SZ-A effectively reduced the release of the pro-inflammatory mediator C-X-C motif chemokine ligand (CXCL)-10, alleviated macrophage infiltration, and regulated M1/M2 macrophage polarization, thereby effectively suppressing the inflammatory response. It is therefore hypothesized that extracts derived from mulberry leaves, or branches hold significant potential for regulating BCAA metabolism, mediating macrophage polarization, and improving inflammatory responses in diabetes.

### 4.8 Probiotics/gut microbiota intervention

In recent years, the role of probiotics in regulating the gut microbiota and its metabolites has become a hot topic. BCAAs are metabolized by gut microbes and drive lipogenesis through signaling pathways such as mTORC1-PPARγ; elevated BCAA levels in the circulation subsequently induce obesity and IR (Cui et al., 2025). Probiotics can effectively modulate beneficial gut microbiota, reducing gut microbiota-mediated BCAA synthesis or promoting BCAA catabolism, thereby lowering BCAA levels and alleviating systemic inflammatory responses and IR (Qiao et al., 2022; Zhang et al., 2026). Chen et al. (2025) found that the probiotic (*Parabacteroides johnsonii*) can reshape the gut microbiota and enhance the conversion of BCAAs to isobutyric acid (IBA); IBA improves gut barrier function, suppresses inflammation, and reduces the expression of pro-inflammatory factors such as TNF-α, IL-1β, and MCP1 in the colon, liver, and adipose tissue by inhibiting HDAC3 and activating the FGF1 pathway, thereby correcting dysregulation of glucose and lipid metabolism. Furthermore, a study by Yoshida et al. (2021) found that *Bacteroides dorei* and *Bacteroides vulgatus* can improve metabolic disorders by enhancing the catabolism of BCAAs in brown adipose tissue (BAT). By reducing BCKDHA phosphorylation and increasing BCKD activity, these two species significantly enhance BCAA oxidation capacity in BAT, thereby lowering plasma and fecal BCAA levels. Concurrently, these bacteria reduced the infiltration of F4/80^+^ macrophages into BAT, restrained macrophage activation, and decreased the expression of pro-inflammatory mediators, including TNF-α, IL-6, and MCP-1, thereby alleviating both local and systemic inflammation. Meanwhile, they upregulate the thermogenic gene UCP1 in BAT, improving glucose tolerance and insulin sensitivity. Additionally, body weight, hepatic lipid deposition, serum triglyceride and cholesterol levels are lowered, achieving effective regulation of glucose and lipid metabolic homeostasis. Studies by Zhang et al. (2025c) indicate that Clostridium butyricum 337279 (C.B.) can downregulate BCAA biosynthesis in the gut microbiota, thereby reducing plasma BCAA/BCKA levels. Concurrently, C.B. reduces levels of inflammatory factors such as TNF-α, IL-1β, and IL-6, inhibits the infiltration of F4/80^+^ macrophages in the liver and white adipose tissue, and improves glucose tolerance and insulin sensitivity in obese mice.

**Table 2** summarizes dietary, pharmacological, natural compound-based, and gut microbiota-targeted strategies that modulate BCAA metabolism, reshape macrophage polarization, and alleviate diabetic metabolic inflammation.

**Table 2:** Intervention strategies targeting BCAA metabolism to regulate macrophage polarization and diabetic metabolic inflammation.

Representative intervention	Effects on BCAA metabolism	Effects on macrophage polarization / inflammation	Metabolic benefits in diabetes or insulin resistance	References
Low-BCAA diet and Calorie restriction	Reduces exogenous BCAA load and circulating BCAA exposure, thereby lowering BCAA-associated metabolic stress.	Prevents high-fat diet-induced loss of M2 macrophages and reduces adipose macrophage infiltration and inflammatory cytokines such as TNF-α, IL-1β, and IL-6.	Improves glucose tolerance and insulin sensitivity, partly independent of calorie restriction, although some effects may be modest.	(Fontana et al., 2016; Cummings et al., 2018; Liu et al., 2022; Zhang et al., 2025a)
Exercise intervention	Enhances systemic leucine turnover, upregulates BCKDH-related genes and SLC25A44, and reduces adipose BCKDK expression, thereby promoting BCAA/BCKA catabolism.	Reduces M1-like macrophage accumulation, IL-1β, and iNOS, increases IL-10 and M2-related signaling, and inhibits miR-221-3p/JAK/STAT-mediated M1 polarization.	Improves insulin sensitivity and skeletal muscle IR and alleviates metabolic inflammation.	(Glynn et al., 2015; Lee et al., 2021; Li et al., 2022, 2025; Huang et al., 2024)
BCAA catabolic activator: BT2	Inhibits BCKDK-mediated BCKDH/BCKDC phosphorylation, activates BCKDH, and enhances BCAA/BCKA oxidation .	Suppresses M1 polarization, reduces Iba1^+^ macrophage infiltration, and lowers TNF-α and CCL2 release.	Lowers plasma and tissue BCAA/BCKA levels and improves insulin sensitivity, glucose tolerance, hepatic steatosis, and liver injury.	(Tso et al., 2014; Zhou et al., 2019a; Bollinger et al., 2022)
PPARγ agonist: Rosiglitazone	Reduces serum BCAA levels and increases BCAT and BCKDH activity in adipose tissues.	Activates macrophage PPARγ, inhibits M1 polarization, and promotes M2-like anti-inflammatory polarization.	Improves glucose intolerance and IR in obese models .	(Odegaard et al., 2007; Blanchard et al., 2018; Rohm et al., 2024)
Berberine	Reduces BCAA-producing gut bacteria, decreases BCKDHA phosphorylation and BCKDK levels, and promotes hepatic and adipose BCAA catabolism.	Inhibits MAPK/ROS and AKT1/SOCS1/NF-κB signaling, reducing M1 markers and pro-inflammatory cytokines including TNF-α, IL-1β, IL-6, MCP-1, and iNOS .	Improves glucose tolerance and IR and reduces liver and adipose inflammatory infiltration.	(Jeong et al., 2009; Liu et al., 2018; Neag et al., 2018; Yue et al., 2019; Song et al., 2020; Wang et al., 2024a)
Ginsenoside Rb1	Regulates gut microbiota and BCAA metabolism in high-fat diet-induced IR.	Inhibits STING-mediated macrophage activation, suppresses macrophage migration, upregulates PPARγ, and blocks NF-κB signaling.	Improves insulin signaling, IR, and metabolic inflammation .	(Zhou et al., 2019b; Hou et al., 2021; Yang et al., 2021; Ding et al., 2023; Wang et al., 2024b)
Mulberry leaf / mulberry twig extracts	Shows potential to modulate BCAA metabolism.	Inhibits TLR2/MyD88/NF-κB signaling, lowers TNF-α and CXCL10, and reduces macrophage infiltration and M1/M2 imbalance .	Restores InsR/IRS1 signaling and improves chronic inflammation and IR in T2DM models.	(Cummings et al., 2018; Tian et al., 2019; Ma et al., 2022; Liu et al., 2023; Zheng et al., 2023)
Probiotics / gut microbiota modulation	Reduces microbial BCAA biosynthesis or promotes BCAA catabolism; P. johnsonii enhances BCAA conversion to isobutyric acid, B. dorei and B. vulgatus activate BAT BCAA oxidation, and C.B. lowers plasma BCAA/BCKA levels.	Reduces F4/80^+^ macrophage infiltration, macrophage activation, and inflammatory factors including TNF-α, IL-1β, IL-6, and MCP1.	Improves gut barrier function, glucose tolerance, insulin sensitivity, body weight, hepatic lipid deposition, and serum lipid profiles .	(Yoshida et al., 2021; Qiao et al., 2022; Chen et al., 2025; Cui et al., 2025; Zhang et al., 2025b, 2026)

## 5 Conclusions and outlook

Based on the above findings, we conclude that BCAA metabolic dysregulation is a characteristic metabolic abnormality of diabetes. The BCAA-macrophage-metabolic inflammation axis serves as a key link connecting metabolic dysregulation, immune imbalance, IR, and the progression of diabetes. Abnormal BCAA catabolism can drive an imbalance in M1/M2 macrophage polarization through immunometabolic reprogramming. The specific mechanisms are as follows: ① Excessive BCAAs (particularly leucine) activate the mTORC1-HIF-1α signaling axis, significantly upregulating key enzymes of glycolysis and promoting the reprogramming of macrophages toward an M1-like pro-inflammatory metabolic phenotype; ② Impaired BCAA catabolism disrupts the tricarboxylic acid cycle and inhibits OXPHOS. It also compromises fatty acid oxidation and mitochondrial function, both of which are required for M2-like polarization, thereby suppressing the anti-inflammatory and reparative phenotype of macrophages. ③ The massive accumulation of BCAA degradation intermediates—BCKA, 3-HIB, and BCAC—induces lipotoxic stress and activates macrophages, further amplifying pro-inflammatory signaling; ④Excessive BCAA/BCKA disrupts mitochondrial homeostasis, triggering a burst of mitochondrial ROS, which in turn induces endoplasmic reticulum stress. Together, these factors activate the NLRP3 inflammasome, promoting the maturation and release of pro-inflammatory factors such as IL-1β, thereby amplifying the M1 pro-inflammatory response; ⑤ BCAAs continuously activate classic inflammatory pathways such as IFNGR1/JAK1/STAT1 and NF-κB, stably maintaining the M1-like polarization state of macrophages, ultimately leading to an imbalance in M1/M2 polarization and driving diabetes-related metabolic inflammation. Therefore, BCAA metabolic dysregulation serves as a key upstream driver of abnormal immune-metabolic reprogramming, polarization imbalance, and persistent chronic inflammation in diabetic macrophages. Targeted regulation of BCAA metabolism, including low-BCAA diet, nutritional restriction, exercise intervention, BCAA catabolism activators, PPARγ agonists, berberine, ginsenoside Rb1, mulberry leaf and twig extracts, as well as probiotics and gut microbiota, can effectively restore macrophage polarization balance and mitigate metabolic inflammation, providing a novel multi-target intervention strategy for diabetes.

However, current research on how BCAA metabolic abnormalities drive macrophage polarization imbalance through immunometabolic reprogramming still faces numerous limitations. Existing studies lack systematic validation of the mechanisms by which BCAA metabolic dysregulation leads to immunometabolic reprogramming, which in turn drives M1/M2 macrophage polarization and influences diabetes-related metabolic inflammation. Furthermore, Furthermore, it remains unclear whether BCAAs determine macrophage phenotype and function through epigenetic modifications, non-coding RNA-mediated regulation, or metabolite–protein interactions. Additionally, current research is largely confined to animal experiments and cellular models, lacking support from large-scale population cohort studies and randomized controlled clinical trials. In particular, there remains a limited number of drugs capable of specifically targeting BCAA metabolism and precisely regulating macrophage polarization, which restricts effective interventions for diabetes-related metabolic inflammation. It is hoped that in the future, large-scale randomized controlled trials involving interventions such as low-BCAA diets, BCKDK inhibitors, and berberine can be conducted to clarify the therapeutic effects of targeting BCAA metabolism on diabetes-related metabolic inflammation. Furthermore, we aim to conduct in-depth analyses of the epigenetic, non-coding RNA, and metabolite-protein interactions involved in BCAA regulation of macrophage polarization. At the same time, we are developing BCAA metabolism-targeted drugs with macrophage specificity to reduce systemic side effects and enhance therapeutic precision. We will further explore strategies for combining these BCAA metabolism-targeted drugs with existing antidiabetic medications, with the aim of improving glucose and lipid metabolism while alleviating chronic metabolic inflammation, thereby enhancing the overall therapeutic efficacy of diabetes treatment.
